# Behavioural Susceptibility Theory: Professor Jane Wardle and the Role of Appetite in Genetic Risk of Obesity

**DOI:** 10.1007/s13679-017-0247-x

**Published:** 2017-02-24

**Authors:** Clare H. Llewellyn, Alison Fildes

**Affiliations:** 1 Department of Behavioural Science and Health, University College London, London, UK; 2 School of Psychology, University of Leeds, Leeds, UK

**Keywords:** Obesity, Genetic, Heritability, Appetite, Eating behaviour, Behavioural susceptibility

## Abstract

**Purpose of Review:**

There is considerable variability in human body weight, despite the ubiquity of the ‘obesogenic’ environment. Human body weight has a strong genetic basis and it has been hypothesised that genetic susceptibility to the environment explains variation in human body weight, with differences in appetite being implicated as the mediating mechanism; so-called ‘behavioural susceptibility theory’ (BST), first described by Professor Jane Wardle. This review summarises the evidence for the role of appetite as a mediator of genetic risk of obesity.

**Recent Findings:**

Variation in appetitive traits is observable from infancy, drives early weight gain and is highly heritable in infancy and childhood. Obesity-related common genetic variants identified through genome-wide association studies show associations with appetitive traits, and appetite mediates part of the observed association between genetic risk and adiposity.

**Summary:**

Obesity results from an interaction between genetic susceptibility to overeating and exposure to an ‘obesogenic’ food environment.

## Introduction

A century ago, no more than 1 in 20 of the population were obese, the figure had risen to more than 1 in 10 by 1990, and currently it is 1 in 4 (Health Survey for England). Combining overweight and obesity together, almost three in four UK adults carry too much body fat (Health Survey for England) and one in three children (National Child Measurement Programme 2014/15). The rapid increases in weight are widely believed to have been caused by changes in lifestyle and the food supply, creating what is often called an ‘obesogenic’ environment. Changes in transportation and mechanisation have reduced the amount of energy expenditure required to perform a variety of tasks on a day-to-day basis [1]. At the same time, the range of sedentary screen-based entertainments has also increased, further reducing activity levels [1]. Developments in food production, processing, storage and preparation have resulted in highly palatable and energy-dense foods becoming more accessible and cheaper [2]. The food environment does not force us to overeat, but the opportunities and incentive structures have changed making it easy—consciously or unconsciously—to end up in positive energy imbalance, leading to weight gain.

However, despite the ubiquity of the ‘obesogenic’ environment, not everyone is overweight. Even within the same family, there can be striking differences between the weights of siblings living in the same household. It is clear that there is substantial variation in susceptibility to the ‘obesogenic’ environment, and the basis of this variation has been of great interest to obesity researchers. One hypothesis put forward is that differential susceptibility to obesity has a genetic basis, and excess weight gain arises from a combination of genetic risk *and* environmental exposure. Professor Jane Wardle developed the behavioural susceptibility theory (BST) to explain how this interaction between genetic risk and environmental exposure results in weight gain, proposing genetically determined differences in appetite as the mediating mechanism [3].

BST has important implications for the prevention and management of obesity and public health policy. Genes set our potential for becoming obese, but the environment determines the outcome. George Bray put it well when he said: ‘Genes load the gun, and the environment pulls the trigger’ [4]. This review summarises evidence for gene-environment interplay in human body weight and the role of appetite as a mediator of genetic risk. The implications of BST for prevention, management and policy are discussed.

## Gene-Environment Interplay in Human Body Weight

For over a century, researchers have studied twins to estimate the extent to which variability in human body weight is influenced by genes and the environment. Twin designs allow researchers to establish this because identical twins (monozygotic, MZs) are 100% genetically identical, while non-identical twins (dizygotic, DZs) share on average only 50% of their segregating genes, but importantly, both types of twins share their environments to a very similar extent (e.g. they are gestated in the same mother for the same period of time, grow up in the same household and are of the same socioeconomic status). Resemblance between both types of twins can therefore be compared to estimate genetic and environmental influence on body weight (or any other measured characteristic of interest). Greater similarity between the body weights of MZ versus DZ pairs indicates a genetic contribution to body weight. The statistic derived is ‘heritability’ which quantifies the proportion of variation in a trait (e.g. body weight) attributable to genetic variation and ranges from 0% (genetic variation plays no role in explaining variability in a trait) to 100% (genetic variation entirely explains variability in a trait). But twins also provide important insight into different sources of environmental influence, because aspects of environmental variation are partitioned out into those completely shared by two twins in a pair, contributing to within pair similarity (the ‘shared environment’, e.g. maternal gestational weight gain), and aspects that are unique to each individual twin, contributing to differences within pairs (the ‘non-shared environment’, e.g. illness).

Nearly a century of research with twins has established that human body weight (indexed using body mass index (BMI)) is highly heritable [5, 6], but heritability estimates vary considerably. In a review of 32 studies [7], the median heritability estimate was 73% but ranged from 32 [8] to 90% [9]. Gene-environment interplay seems to explain much of this variability. The review found that heritability estimates were higher in populations living in more ‘obesogenic’ environments characterised by those with a higher average BMI and those from countries with a higher average gross domestic product. Obesity prevalence tends to be higher in families of lower socioeconomic status (SES) in Western countries, making lower SES another marker of a more ‘obesogenic’ environment. A large longitudinal study of 16,646 Dutch twin pairs from 1 to 20 years of age found significantly higher genetic variance in BMI for children with less educated parents and lower genetic variance in children with better educated parents [10]. The modifying effect of SES on genetic variance in BMI has been replicated in adult populations, using their own education level [11, 12].

Another way to capture exposure to the ‘obesogenic’ environment is to estimate the heritability of BMI at a particular age, by year-of-birth, the assumption being that those born later have spent a greater proportion of their life in a more ‘obesogenic’ environment. A large Swedish study of around 2000 twin pairs and 115,000 siblings born between 1951 and 1983 showed that genetic variance in BMI at age 18 was higher for those born later, and by implication living in a more ‘obesogenic’ environment [13]. Together, these findings suggest that genetic influence depends to some extent on environmental exposure—the more ‘obesogenic’ the environment, the greater the genetic influence on weight.

The Collaborative Project of Development of Anthropometrical Measures in Twins (CODATwins) is a large-scale initiative of pooled twin studies that includes 434,723 twin individuals (201,192 twin pairs) from 48 studies across 22 countries [14]. This study showed profound developmental variation in genetic influence, which increased from a moderate 40% at 4 years of age to 75% by 19 years of age [15]. At the same time, an important influence of the *shared* environment was observed in middle childhood, but decreased steadily with age (in parallel with increasing genetic risk) such that by 15 years of age, it had disappeared entirely. While it may seem counterintuitive for genetic influence to strengthen (and shared environmental influence to diminish) as children mature, the gain in independence means increasing exposure to the wider ‘obesogenic’ environment. This observation is consistent with a model of gene expression depending on environmental exposure.

The high heritability estimates observed for human body weight made it a promising phenotype for genome-wide association studies to identify common genetic variants contributing to variation. A variant in the fat mass and obesity-associated gene (FTO) was the first to be discovered and has the largest effect size of all known variants to date [16]. Adults of average height who carry two copies of the high-risk variant (homozygotes) are approximately 3 kg heavier than adults who carry two copies of the low-risk variant. FTO was an important discovery, not only because about half of the population carries at least one of the high-risk variants, but also because the effect size was large enough for researchers to explore its mechanisms. Currently, 97 common variants have been robustly associated with body mass index (BMI) in genome-wide meta-analyses [17••]. These can be aggregated into a composite genetic risk score that is quantitatively associated with BMI, explaining approximately 3% of variation among adults and children.

As is observed with twin studies, the environment also modifies the impact of measured genetic risk of obesity. In a large European sample of children (*n* = 4406), low parental socioeconomic position accentuated the effect of FTO on adiposity [18], and in an adult sample, the higher risk variant of FTO was only associated with obesity risk in participants with no university education [19]. Age-related increases in the magnitude of the association between measured genetic risk of obesity (indexed using FTO and composite genetic risk scores) and BMI have also been observed [20–23], in line with patterns of heritability from twin data. The strongest evidence yet has come from a recent study showing that the association between measured genetic risk of obesity (using a composite score) and BMI was significantly larger for more recent birth cohorts, i.e. those with who had had greater overall exposure to the ‘obesogenic’ environment [24].

Together, these studies provide convincing evidence that genetic risk of obesity depends on exposure to an ‘obesogenic’ environment. The question is *how* ‘obesogenic’ environments accentuate the effects of genes to maximise genetic expression. Jane Wardle proposed that the basis of the interaction is inherited differences in appetite, which make some individuals more likely to overeat in response to the many opportunities offered by the current food environment, so-called *behavioural susceptibility theory* (BST) [3].

## Behavioural Susceptibility Theory: Appetite Mediates Genetic Risk of Obesity

BST hypothesises that genes influence weight at least partly through their effects on appetite—i.e. there is variation in appetite that has a strong genetic basis, and variation in appetite causes differences in body weight. The idea that appetite plays a causal role in obesity is not new; it was first proposed by Stanley Schachter in 1968 [25]. In a series of innovative experiments, he observed that obese adults ate significantly more than healthy weight adults when the food on offer was highly palatable, but showed no difference in intake in response to bland foods. At the same time, obese adults did not show the same compensatory down-regulation of food intake following a high-calorie snack as normal-weight adults, indicating blunted satiety (fullness) or an overriding of satiety. Schachter came up with externality theory to explain these observations. He proposed that obese individuals have two distinct aberrations in appetite regulation that lead to overeating; they are overly responsive to highly palatable food cues (wanting to eat (or eat more) in response to the sight, smell and taste of palatable food) and are under-responsive to internal satiety mechanisms (fullness).

**Fig. 1:**
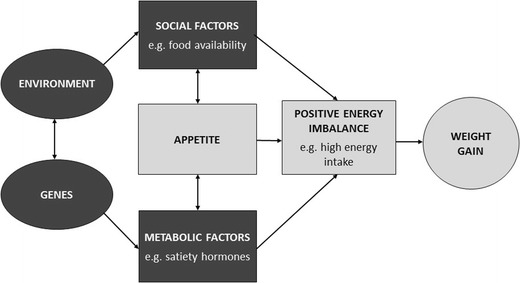
How appetite mediates the interaction between genetic susceptibility to obesity and environmental exposure. Individuals who inherit a set of genes that bestow greater responsiveness to external food cues and/or lower sensitivity to satiety are more likely to overeat in response to an ‘obesogenic’ food environment, and to gain excessive weight. Obesity therefore results from a combination of genetic susceptibility to overeating and exposure to an ‘obesogenic’ food environment

Wardle was the first to make the link between these appetitive characteristics identified by Schachter and genetic risk of obesity. She developed the BST in which she hypothesised that genes influence weight at least partly via biological mechanisms that control appetite regulation [26]. The BST explains how human body weight can have *both* genetic *and* environmental drivers at the same time and why genetic expression on weight is likely to be stronger in more ‘obesogenic’ environments (see Fig. 1). Individuals who are genetically predisposed to be highly responsive to food cues are more likely to overeat in an environment in which food cues pervade every aspect of daily living. Those predisposed to weaker satiety signals are more likely to overeat in response to larger portion sizes and multiple opportunities to eat.

### Development of Psychometric Measures of Appetite for Children

In order to test the BST, Wardle needed to measure these appetitive characteristics in samples large enough to establish reliable associations with weight, demonstrate generalisability and estimate genetic influence. Laboratory-based measures of appetite provide unparalleled detail, but the time and expense incurred prohibit measuring eating behaviour objectively in large samples. Another limitation is that only a single ‘snapshot’ of eating behaviour is captured, and behaviour is subject to any extraneous factors at play during the time of testing. The unfamiliar setting and not liking the test food can be particularly problematic. Wardle also saw limitations to studying the BST in adults and considerable advantages to undertaking research with infants and children. Adults with longstanding obesity may have abnormalities in appetite as a result of physiological changes caused by the excess weight itself, and overweight adults are often dieting. Young children are unlikely to be dieting and are too young to have experienced long-term effects of chronic obesity. Studying infants prospectively into childhood provides the opportunity to study the cause-effect relationship between appetite and weight as it starts to emerge.

Wardle therefore developed the Child Eating Behaviour Questionnaire (CEBQ), a comprehensive parent-report psychometric measure of a range of children’s appetitive characteristics, to test the BST in large samples of children [27]. While standardised psychometric measures lose the objectivity of laboratory-based observations, they have the advantage of characterising habitual eating behaviour over many meals and situations—in this respect, such measures capture the enduring appetitive ‘trait’ rather than a ‘state’ of hunger or fullness at the time of testing. Young children lack the comprehension skills or self-awareness to answer questions about their own behaviour, but parents tend to know them very well, arguably making them the most accurate informants of their children’s behaviour. Parent report is of course subjective and has the potential for bias, but the CEBQ has been validated against objectively measured eating behaviour [3].

The CEBQ is a comprehensive measure of all observable aspects of children’s eating behaviour hypothesised to play a causal role in overweight or to protect against overweight. Parents respond to items that describe a range of eating behaviours using a five-point frequency scale (‘never’, ‘rarely’, ‘sometimes’, ‘often’, ‘always’) to indicate the frequency with which their child demonstrates each behaviour. Two scales characterise Schachter’s behavioural measures of responsivity to food cues and are hypothesised to predispose to overweight: ‘food responsiveness’ measures a child’s tendency to want to eat when prompted by palatable foods (e.g. ‘even if my child is full up, s/he finds room for his/her favourite food’), and ‘enjoyment of food’ captures the subjective reward experienced while eating (e.g. ‘my child enjoys eating’); higher scores on these scales indicate a more avid appetite. Two other scales characterise Schachter’s behavioural measures of responsivity to internal satiety cues and are hypothesised to protect against overweight: ‘satiety responsiveness’ measures a child’s fullness threshold (e.g. ‘my child gets full before his/her meal is finished’) and ‘slowness in eating’ captures the pace with which a child finishes a meal, with higher eating speed hypothesised to outpace biological satiety mechanisms (e.g. ‘my child takes more than 30 min to finish a meal’); higher scores on these scales indicate better appetitive control. Two scales measure the tendency to either under- or overeat in response to negative emotions: ‘emotional overeating’ is thought to predispose to overweight (e.g. ‘my child eats more when worried’), and ‘emotional undereating’ is hypothesised to protect against overweight (e.g. ‘my child eats less when upset’). ‘Food fussiness’ assesses the tendency for a child to be highly selective about what they will agree to eat and captures both refusal to try unfamiliar foods (termed ‘neophobia’, e.g. ‘my child refuses new foods at first’) as well as pickiness about the textures and tastes of familiar foods (e.g. ‘my child is difficult to please with meals’). Fussiness is a common characteristic of children who fail to thrive, because they often eat too little; this trait may therefore offer some protection against overweight.

The CEBQ scales have good internal and external reliability [27] and show strong tracking from early to late childhood [28] indicating that they characterise fairly stable traits that persist over development. The CEBQ has also been adapted to measure appetitive traits during infancy (the Baby Eating Behaviour Questionnaire, BEBQ [29]), and more recently in adulthood (the Adult Eating Behaviour Questionnaire, AEBQ [30]). The BEBQ measures four of the same appetitive traits during the period of exclusive milk feeding, before any solid food has been introduced: ‘food responsiveness’, ‘enjoyment of food’, ‘satiety responsiveness’ and ‘slowness in eating’. The AEBQ is a self-report version of the CEBQ for adults, capturing largely the same appetitive traits as the CEBQ: ‘food responsiveness’, ‘enjoyment of food’, ‘satiety responsiveness’, ‘slowness in eating’, ‘emotional overeating’, ‘emotional undereating’ and ‘food fussiness’, but with the addition of a ‘hunger’ scale to capture experienced levels of hunger (arguably only possible via self-report) [29]. Together, these three measures enable assessment of eating behaviour across the life course from infancy (BEBQ) and childhood (CEBQ) into adulthood (AEBQ).

The development of these psychometric measures of appetite has led to the emergence of an extensive literature exploring the relationship between appetite and weight in large population-based samples, the genetic basis of appetite and the role of appetite in mediating genetic risk of obesity. The majority of research has focused on the scales that characterise the appetitive traits relating to Schachter’s externality theory, ‘food responsiveness’ and ‘enjoyment of food’ (indexing hyper-responsiveness to external food cues) and ‘satiety responsiveness and slowness in eating’ (indexing blunted responsiveness to internal satiety cues).

### Variation in Appetitive Traits Drives Weight Gain

A wealth of cross-sectional research with the CEBQ has established almost without exception that ‘food responsiveness’ and ‘enjoyment of food’ are positively and ‘satiety sensitivity’ and ‘slowness in eating’ are negatively associated with measures of adiposity, in different samples of children ranging from 3 to 13 years of age [31–36]. Importantly, studies have also demonstrated that these appetitive traits influence weight across the whole spectrum in a graded fashion. They do not simply distinguish the clinically obese from the ‘healthy weight’ children, but explain more subtle variation in weight as well, e.g. between children at the lower versus higher end of ‘healthy weight’ [31, 32, 34–36].

This research provides support for the BST insofar as greater adiposity is characterised by distinctive eating behaviours that predispose to overeating, but cross-sectional data cannot provide any insight into the direction of the relationship between appetite and weight. Establishing the cause-effect relationship is not a straightforward task. It is not possible to randomise individuals to be more or less food responsive or satiety sensitive, and then examine the impact on weight. An alternative approach is to use prospective data to establish if variation in appetite predicts weight gain better than variation in weight predicts appetite change. The BST also hypothesises that these appetitive traits, like body weight itself, have a genetic basis.

Wardle established a large population-based prospective birth cohort of 2402 infant twin pairs, Gemini [37], to explore genetic and environmental influence on early growth, with a focus on behavioural pathways. Two design features ensure that the Gemini cohort is well placed to examine the validity of the BST: (i) it is a prospective birth cohort allowing the direction of the relationship between appetite and weight to be tracked as it starts to emerge in early life and (ii) the twin design allows the genetic and environmental influence on appetite to be explored.

Gemini was the first study to examine the (bidirectional) prospective relationships between appetite and weight from birth. Data strongly supported the hypothesis that variation in appetite at 3 months was driving early weight gain from 3 to 15 months, not the other way around (weight variation at 3 months did not predict appetite change from 3 to 15 months) [38]. A follow-up study strengthened this finding, by comparing the growth trajectories from 3 to 15 months of twin pairs discordant for ‘food responsiveness’ (*n* = 121 pairs) and ‘satiety responsiveness’ (*n* = 172 pairs). This design enabled an investigation of the relationship between appetite and weight gain while controlling for important environmental confounders that are completely shared by twin pairs (e.g. maternal pre-pregnancy weight, gestational weight gain, maternal diet during pregnancy, SES). The weights of the twin pairs diverged progressively such that by 15 months of age, there was a 1-kg difference, equating to a 10% difference in body weight [39••]. The only subsequent prospective study of 210 infants from Singapore also found that higher ‘food responsiveness’ and lower ‘satiety responsiveness’ (and ‘slowness in eating’) were associated with greater infant weight gain [40].

### Appetitive Traits Mediate Genetic Influence on Weight

Using the BEBQ, Gemini has also established the relative contribution of genetic and environmental influence to variation in appetitive characteristics during the earliest period of life, when infants are still exclusively milk-fed [41]. Heritability was substantial for each of ‘enjoyment of food’ (53%), ‘food responsiveness’ (59%), ‘satiety responsiveness’ (72%) and ‘slowness in eating’ (84%). This finding is striking, given that the BEBQ captures variation in appetite for milk only—even very early in life infants vary considerably in their appetite, and this variation is both associated with weight gain and is genetically based. A follow-up study to quantify the extent to which there is genetic overlap between appetite and weight at 3 months found that approximately one third of the genetic influences underlying 3-month weight are the same as those underlying appetite, supporting the hypothesis that genes influence weight partly through effects on appetite [42].

The infant study in Gemini followed only one previous examination of the heritability of ‘enjoyment of food’ and ‘satiety responsiveness’ in a very large population-based sample of 10-year-old twin children (*n* = 5435 pairs) from the Twins Early Development Study (TEDS), also conducted by Wardle [43]. The heritability estimates observed in older children were of the same magnitude as those observed in infancy for both ‘enjoyment of food’ (75%) and ‘satiety responsiveness’ (63%). Eating speed, measured objectively in a subsample of the children (*n* = 254), also showed high heritability (62%) [44].

The recent discovery of common genetic variants associated with human body weight has opened up new avenues for detailed examinations of the mechanisms involved, and the likely role of appetite. Shortly after the discovery of FTO, Wardle and her colleagues [45] used data from TEDS to show that 10-year-old children who carried at least one copy of the lower risk variant (TT or AT) were significantly more satiety sensitive than those carrying two copies of the higher-risk version (AA). This effect remained after adjustment for BMI, indicating that FTO is influencing body weight via impacting satiety sensitivity. This study was replicated using a behavioural measure of satiety sensitivity in a subsample of the children at 5 years of age [46] and shown independently by Cecil and colleagues [47]. A more recent study [48••] showed that a composite genetic risk score both *with* and *without* FTO was associated with ‘satiety responsiveness’ in the TEDS children at 10 years of age, and mediated part of the association between the genetic risk score and adiposity, indicating that FTO *and* other variants are affecting adiposity partly via mechanisms that regulate satiety.

In three other large independent samples of adults (*n* = 4632, *n* = 1231 [49]; *n* = 3852 [50]), questionnaire measures of ‘uncontrolled eating’ (a measure of extreme hunger and eating trigged by external food cues) and ‘emotional eating’ were also associated with genetic risk of obesity, and these appetitive traits mediated part of the association between the genetic risk score and adiposity [49]. Gene expression studies have also strongly supported an appetitive pathway insofar as many of the common risk variants are in or near genes that are highly expressed in the hypothalamus and pituitary gland, key sites of central appetite regulation [17••].

The magnitudes of the associations between known obesity-related genetic variants and appetitive traits (and body weight itself) are often disappointingly small, but identifying the mechanisms is nevertheless an important endeavour. Establishing causal pathways can guide researchers towards targeted interventions to reduce obesity risk. The evidence for genetic risk of obesity operating (at least partly) via appetitive mechanisms suggests that behavioural processes may serve as useful intervention targets.

### Behavioural Expressions of Appetitive Traits and Weight Gain

A key question of interest has been *how* these genetically determined appetitive traits lead to weight gain in response to the current ‘obesogenic’ environment. In other words, what are the *behavioural* expressions of these traits that lead to overeating and weight gain in an *everyday* context? Recent research in Gemini has established that greater responsiveness to food cues and blunted satiety sensitivity are characterised by distinctive (different) ‘everyday’ patterns of excess intake, in very young children. When the twins were approximately 2 years old, parents completed 3-day diet diaries for 2203 of the children [51]. These data were used to derive two possible patterns of overeating—consuming a larger average meal size at each eating occasion and eating more frequently throughout the day. Relationships between these intake patterns and appetite (‘food responsiveness’ and ‘satiety responsiveness’ measured using the CEBQ at 15 months) were explored. More food responsive children ate more frequently throughout the day but did not eat a larger amount each time [52•]. On the other hand, children with impaired satiety sensitivity consumed larger average meal sizes each time they ate, but did not eat more frequently throughout the day [52•]. These distinctive patterns of overconsumption make sense given what we know about the interaction between appetite and environmental opportunity. Individuals who are highly responsive to food cues are likely to eat more often in response to an environment where food cues are encountered throughout the day. Those with weaker satiety signals are susceptible to overeating in response to larger portion sizes, because they take longer to feel full (or require more energy or a larger sized portion (higher grams) of food).

Subsequent research showed that consuming larger average meal sizes, but not eating more frequently, was the key driver of excessive weight gain from 2 to 5 years of age. But it is important to understand the relative contribution of meal size and meal frequency to weight gain in older children who have more autonomy over how much *and* how often they eat [53•].

## Conclusions

The BST has revolutionised our understanding of the genetic and environmental drivers of human body weight. It is clear that some individuals face a double onslaught of both biological and environmental pressures. Established psychometric measures have revealed that differences in food cue responsiveness and satiety sensitivity begin to emerge after birth and persist across the life-course. Variation in these appetitive traits has a strong genetic basis and impacts early weight gain profoundly. Research into the BST has shown that individuals who inherit a more avid appetite are more susceptible to taking advantage of the many temptations and opportunities offered by the ‘obesogenic’ food environment and to gain excessive weight as a consequence. The behavioural expressions of an avid appetite are eating too often throughout the day (greater food cue responsiveness) and eating too much each time (weakened satiety sensitivity).

BST points very strongly to the likelihood that obesity rates would diminish should the wider food environment change dramatically. The reality is that large-scale government regulation of the food supply is unlikely to happen in the near future, given that public support for making food less accessible, palatable or affordable would probably be low. In the meantime, the development of pharmacological and behavioural treatments that target over-responsiveness to food cues and impaired satiety might provide an avenue for success. Strategies such as careful portion control and slow eating are already used to circumvent poor satiety responsiveness, and there may be other opportunities to attenuate responsiveness to food cues, such as attention control or self-regulation training.

Given that variation in appetite is observable and measurable from early postnatal life, this might provide a useful marker of obesity risk for public health obesity prevention initiatives. Wardle’s work on the BST has also provided a firm conceptual framework for the development and testing of early life interventions to attenuate food cue responsiveness and upregulate satiety sensitivity.
